# Mechanisms for deNOx and deN_2_O Processes on FAU Zeolite with a Bimetallic Cu-Fe Dimer in the Presence of a Hydroxyl Group—DFT Theoretical Calculations

**DOI:** 10.3390/molecules29102329

**Published:** 2024-05-15

**Authors:** Izabela Kurzydym, Izabela Czekaj

**Affiliations:** 1 Faculty of Chemistry, University of Warsaw, ul. Pasteura 1, 02-093 Warsaw, Poland; chemia@chem.uw.edu.pl; 2 Biological and Chemical Research Center, University of Warsaw, ul. Żwirki i Wigury 101, 01-224 Warsaw, Poland; 3 Faculty of Chemical Engineering and Technology, Cracow University of Technology, Warszawska 24, 31-155 Kraków, Poland

**Keywords:** zeolite, DFT, SCR

## Abstract

In this paper, a detailed mechanism is discussed for two processes: deNOx and deN_2_O. An FAU catalyst was used for the reaction with Cu-Fe bimetallic adsorbates represented by a dimer with bridged oxygen. Partial hydration of the metal centres in the dimer was considered. Ab initio calculations based on the density functional theory were used. The electron parameters of the structures obtained were also analysed. Visualisation of the orbitals of selected structures and their interpretations are presented. The presented research allowed a closer look at the mechanisms of processes that are very common in the automotive and chemical industries. Based on theoretical modelling, it was possible to propose the most efficient catalyst that could find potential application in industry–this is the FAU catalyst with a Cu-O-Fe bimetallic dimer with a hydrated copper centre. The essential result of our research is the improvement in the energetics of the reaction mechanism by the presence of an OH group, which will influence the way NO and NH_3_ molecules react with each other in the deNOx process depending on the industrial conditions of the process. Our theoretical results suggest also how to proceed with the dosage of NO and N_2_O during the industrial process to increase the desired reaction effect.

## 1. Introduction

One of the most important environmental issues is the decomposition of nitrogen oxides (NOx) into harmless N_2_ and H_2_O molecules [1]. Nitrous oxide was identified in the Kyoto Protocol as one of the six greenhouse gases; its Global Warming Potential (GWP) is about 310 times greater than that of carbon dioxide [2] and, due to its ease of undergoing a free radical reaction, it depletes the ozone layer [3]. In addition, NOx, including nitrogen oxide, is a harmful contributor to acid rain and photochemical smog, which damages the ecosystem, disrupts plant vegetation, and has harmful effects on animal and human health [4,5,6].

The best currently available technology for NOx removal is selective catalytic reduction (SCR) [7,8,9,10], which is most commonly carried out in the presence of a reducing agent such as ammonia (NH_3_). In addition, studies show that combining the deNOx process with the deN_2_O process to reduce the N_2_O byproduct can be a very important aspect of increasing the suitability of the SCR process in the nitrogen industry [11,12,13,14].

Due to the different SCR process pathways depending on the substrates, knowledge of the detailed mechanism is essential to improve the process conditions carried out in the industry [15]. Based on previous studies on the mechanism by Bendrich et al. [16], the standard and fast SCR pathway was chosen for the calculations. Also important during the SCR process is the behaviour of the N_2_O molecule involved in the reactions [1,17,18,19]. For this reason, consideration was also given to the deN_2_O process in this publication.

Another important aspect concerning the improvement in the nitrogen oxide reduction process is to find a more efficient catalyst that works over wider temperature ranges than the commonly used vanadium catalyst (V_2_O_5_-WO_3_-TiO_2_) [20,21]. Recently, research into zeolite catalysts with deposited transition metal atoms has been of great interest to scientists [22,23,24,25]. Two metals, copper and iron, deserve special attention. Their properties improve the parameters of the SCR process and, due to the high thermal stability of the zeolites that form the support, they are becoming a very promising alternative to vanadium catalysts [26,27,28,29]. Copper and iron alloys with manganese have been also investigated for SCR and low-temperature SCR reactions [30,31].

Efforts were also made to carry out experiments involving zeolite catalysts with deposited bimetallic structures in a Cu-Fe combination [32,33,34]. These attempts showed great promise, as the catalysts demonstrated very high NO conversion, selectivity to N_2_, and good thermal stability across a wide temperature range (about 230–500 °C) and resistance to the harmful effects of sulphur oxides. On this basis, this solution was proposed using FAU zeolite, which showed interesting properties in previous studies [35,36].

As mentioned earlier, a precise understanding of the reaction mechanism is required to fully explore the SCR process and optimise the conditions of the process [16]. Theoretical calculations allow different variants to be tested in a relatively quick and reasonably low-cost way, allowing a wider view of the entire catalytic system, as well as the investigation of, for example, important intermediates that decompose rapidly and are consequently more difficult to analyse in experimental studies [37]. Theoretical studies have also shown that systems in which metal deposition occurs in the form of an oxygen-bridged dimer between metal atoms (M_1_-O_b_-M_2_) are especially important in the deNOx process [38].

In the present work, a catalytic system consisting of an FAU zeolite with a Cu-Ob-Fe bimetallic dimer is presented, based on the literature reports. Different catalyst variants were used—a hydroxyl group on a bridged oxygen atom, a hydroxyl group on a copper atom, and a hydroxyl group on an iron atom. The mechanism of the deNOx process is presented with reference to the literature [16], as well as two variants of deN_2_O, due to the fact that the order of adsorption of nitric oxide and nitrogen dioxide on the catalyst is important in the further steps of the mechanism. An analysis of the ionicity of the atoms, bond lengths, and bond orders is also presented for a more precise interpretation. All calculations were performed using the DFT method and the results were visualised using the Mercury 4.2.0 software [39].

## 2. Results and Discussion

An analysis of the mechanisms of the deNOx and deN_2_O process was carried out on the three types of systems deposited on the FAU zeolite mentioned in the Section 3. In all cases, the previously developed paths of the various stages of the reaction were used [16,35].

**Figure 1:**
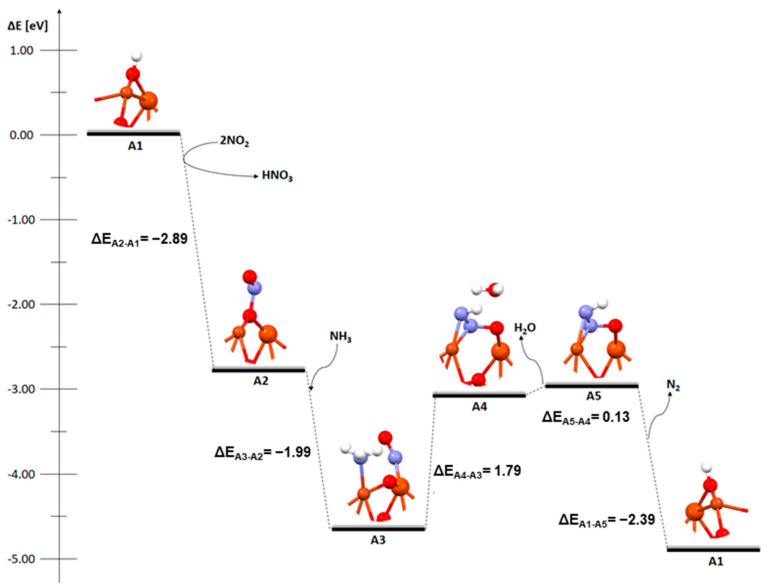
Mechanism of deNOx reaction over FAU catalyst with a Cu-O_b_-Fe dimer with an OH group on the oxygen bridge.

Figure 1 shows the mechanism of the deNOx process on an FAU zeolite structure with a Cu-Fe bimetallic dimer and hydroxyl group on the bridged oxygen.

In the first step, A1-A2, following the concept for the Fast-SCR process according to Bendrish et al. [16], a reaction takes place between two NO_2_ molecules and hydrogen in the OH group. As a result of this reaction, a nitric acid(V) molecule is formed and a NO molecule is attached to the dimer. This stage is strongly exothermic (−2.89 eV). The next step, A3, is the attachment of ammonia. This phase is also exothermic (−1.99 eV). When NH_3_ is attached, NO moves and attaches to the iron atom. In the next two stages, A4 and A5, the transformation of the system towards the formation of non-toxic water and nitrogen molecules and desorption of the formed water molecule are endothermic and their energies are 1.79 eV and 0.13 eV, respectively. In the last stage, A5-A1, desorption of the nitrogen molecule and reconstitution of the initial catalyst structure is exothermic and its energy is −2.39 eV.

**Figure 2:**
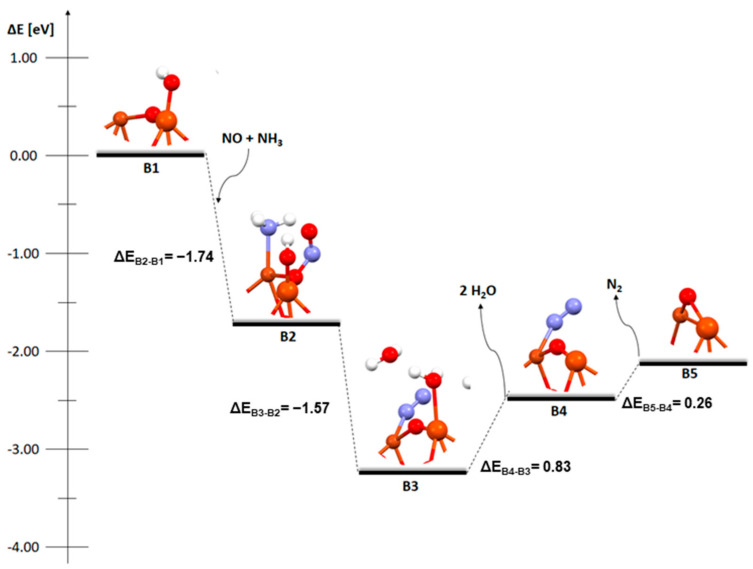
Mechanism of deNOx reaction over FAU catalyst with a Cu-O_b_-Fe dimer with an OH group on iron.

**Figure 3:**
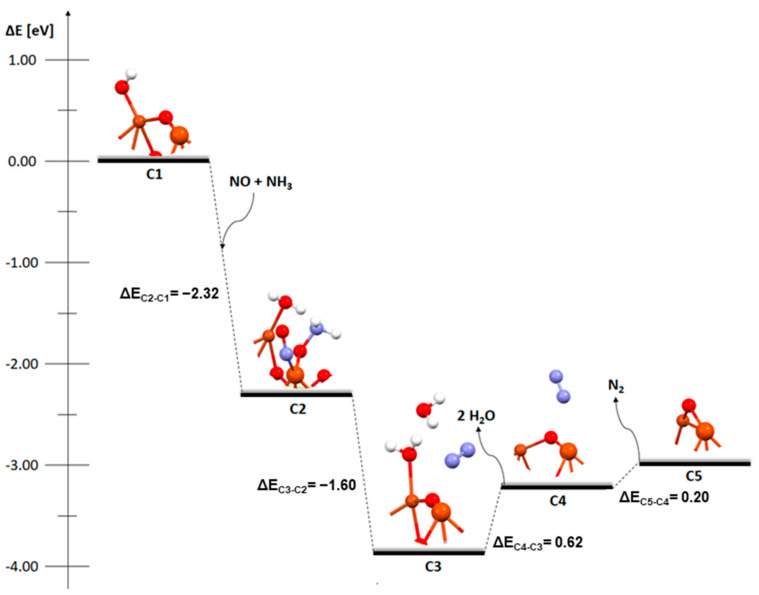
Mechanism of deNOx reaction over FAU catalyst with a Cu-O_b_-Fe dimer with an OH group on copper.

The next two figures (Figure 2 and Figure 3) show the mechanism of the deNOx process carried out on a system where the hydroxyl group occurs on one of the metal atoms in the dimer. In both cases, the initial step is different from the structure with an OH group on the bridged oxygen. There is no reaction between NO_2_ and hydrogen; instead, there is simultaneous coadsorption of nitric oxide and ammonia. The choice of this type of step is also motivated by the proposal of Bendrich et al. [16] and our own research [35,36].

As with the previous structure here, the first two stages, B2-B3, of the process on bimetallic dimer with an OH group on iron are also exothermic. The coadsorption energy of nitric oxide and ammonia is −1.74 eV (Figure 2, B1-B2) and the transformation energy of the system to two water molecules and one nitrogen molecule is −1.57 eV. The next two steps, B3-B5, are the desorption of the two water molecules, which is endothermic with an energy of 0.83 eV, and the desorption of the nitrogen molecule, which requires 0.26 eV. The mechanism ignores the stage of reconstitution of the system to the initial stage, as it is not relevant to the present study.

The next mechanism analysed is the one taking place on a structure with a bimetallic dimer, where the OH group is deposited on copper (Figure 3). As in the previous case, both coadsorption and transformation are exothermic steps and require −2.32 eV and −1.60 eV, respectively. At the C2 step, the attachment of both adsorbates (NO and NH_3_) destabilises the dimer structure and breaks the bond between the bridged oxygen and copper. The next two steps, C3-C5, also show similarity and both are endothermic. The energy required for the desorption of two water molecules is 0.62 eV and for the desorption of a nitrogen molecule is 0.20 eV.

An important similarity is the amount of energy required to desorb the non-toxic decomposition products of nitric oxide, which are water and nitrogen molecules. The energy required to desorb H_2_O and N_2_ is lowest for the catalyst with a bimetallic Cu-Fe dimer and a hydroxyl group deposited on a copper atom. Even though the first two steps proceed with greater efficiency (more energy is released to the environment) in a system with a hydroxyl group on bridged oxygen, the amount of energy subsequently required to convert the system to products is significant, which makes its total efficiency across the process uninteresting from an industrial point of view. Of these three systems, the one with hydrated copper in a bimetallic dimer will be the most efficient.

**Figure 4:**
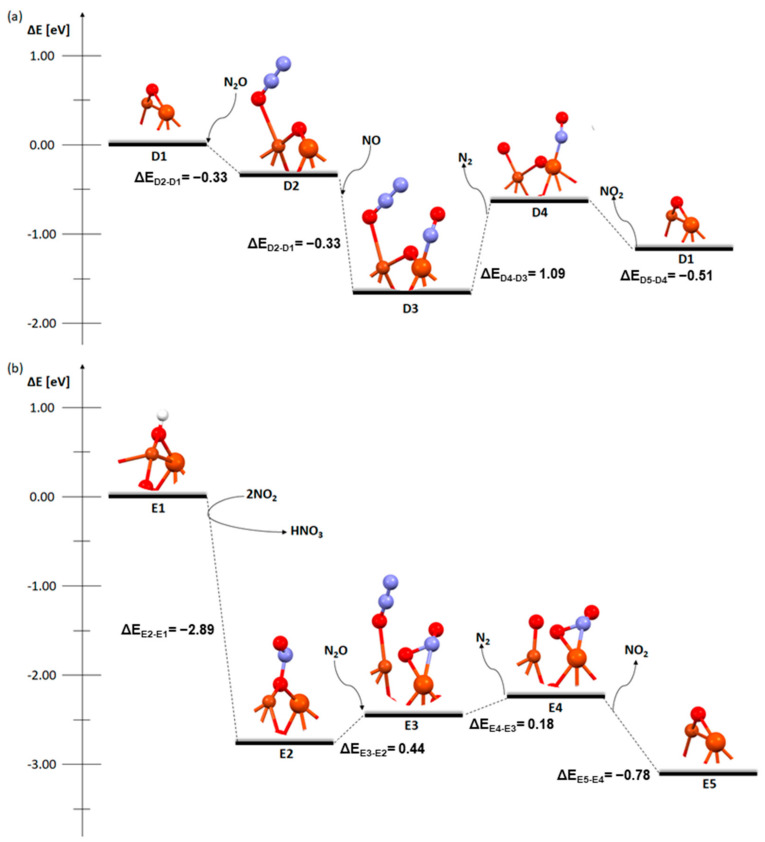
Mechanism of deN_2_O reaction over the FAU catalyst with a Cu-O_b_-Fe dimer with an OH group on the oxygen bridge: (**a**) first absorption of N_2_O and (**b**) first absorption of NO.

**Figure 5:**
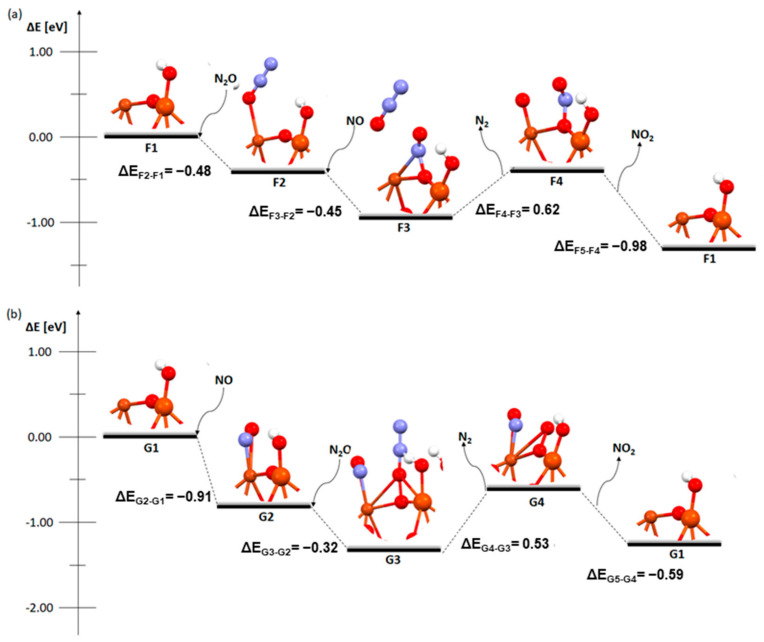
Mechanism of deN_2_O reaction over the FAU catalyst with a Cu-O_b_-Fe dimer with an OH group on iron: (**a**) first absorption of N_2_O and (**b**) first absorption of NO.

**Figure 6:**
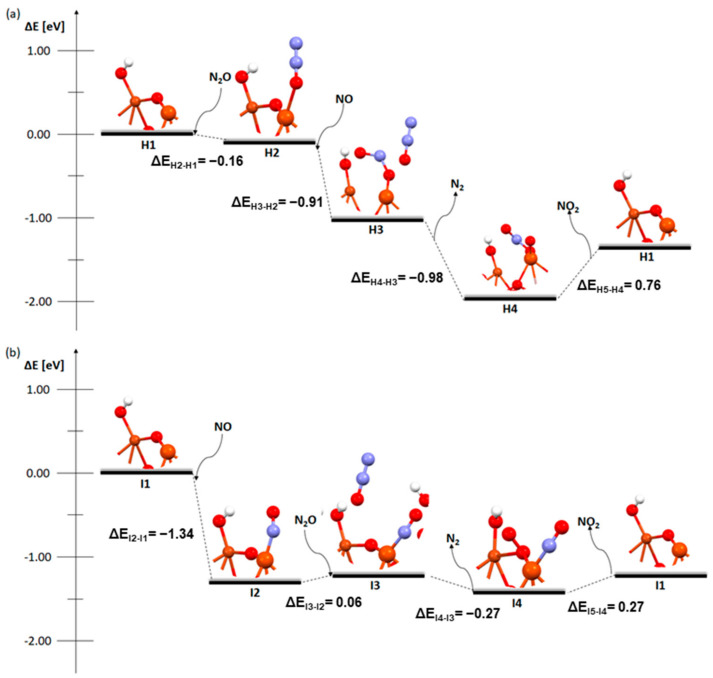
Mechanism of deN_2_O reaction over the FAU catalyst with a Cu-O_b_-Fe dimer with an OH group on copper: (**a**) first absorption of N_2_O and (**b**) first absorption of NO.

The next three figures presented the mechanism of the deN_2_O process on the catalytic systems mentioned earlier (Figure 4, Figure 5 and Figure 6). Two approaches were used to analyze the mechanism of deN_2_O. In the first one (Figure 4a, Figure 5a and Figure 6a), the N_2_O molecule is adsorbed at the beginning. In the other (Figure 4b, Figure 5b and Figure 6b), the same type of reaction as with the deNOx process was used in the initial step (two NO_2_ molecules react with hydrogen on a bridged oxygen atom and HNO_3_ is formed) and nitric oxide adsorbs on the dimer. In N_2_O adsorption on a metallic dimer with an OH group on the bridging oxygen, the hydrogen atom was omitted as it is not needed for the adsorption of N_2_O on the catalyst surface. The N_2_O molecule can directly decompose on zeolite [19].

In Figure 4, which shows the mechanism of the deN_2_O process depending on the order of adsorption of nitric oxide and diazonium oxide, there are significant differences in the first stage. Although both the D1-D2 and E1-E2 stages are exothermic, the adsorption of NO (energy release to the environment of −2.89 eV) is definitely easier. The site and method of adsorption are also different. NO attaches to the bridging oxygen via nitrogen, while N_2_O attaches to one of the metal atoms via oxygen. In the next step, despite the similarity in structures, the bonding is completely different. For Figure 4b in the E3 step, the bond between bridging oxygen and the copper atom is broken, while this does not happen in Figure 4a and the D3 step. In the detachment of the bridging oxygen from the copper, there is an endothermic transition between stages. In the D3 stage, when the dimer structure stays unchanged, the energy of the system is reduced (−1.54 eV) and the transformation is exothermic. The next transition between stages, D3-D4 (desorption of the nitrogen molecule) is endothermic, while the last stage in both cases (desorption of NO_2_) is exothermic (−0.51 eV and −0.78 eV, respectively).

Figure 5 and Figure 6 show two types of the deN_2_O process on structures with a hydroxyl group on one of the metal atoms.

In the mechanism of the deN_2_O process on a bimetallic dimer with a hydroxyl group on the iron atom (Figure 5), there are some similarities with the previous mechanisms. The adsorption of N_2_O and NO in the first stage, F2/G2, is exothermic (−0.48 eV for N_2_O and −0.91 eV for NO), while in both cases (Figure 5a,b—stages F4 and G4), the desorption of the nitrogen molecule is endothermic (0.62 eV and 0.53 eV, respectively) and the desorption of the NO_2_ molecule, F4 and G4, is similar and exothermic (−0.98 eV in the first case and −0.59 eV in the second). Interestingly, despite the fact that the third stage, i.e., coadsorption, proceeds with the same structure and molecules and there is also a release of energy to the environment, the final structures are different. After the adsorption of NO to N_2_O, there is a release of energy of −0.45 eV and NO attaches, while N_2_O detaches and drifts near the reaction system. In the adsorption of N_2_O to NO, the nitric oxide attaches to the copper (not as in the case of F3 to the bridging oxygen) while N_2_O forms a bond with the whole dimer-bridging oxygen and both metals. In this case, −0.32 eV is released.

There are several important changes in Figure 6, where the mechanisms of the deN_2_O process for a Cu-Fe bimetallic catalyst with a hydroxyl group on the copper atom are shown.

First, in the mechanism starting from N_2_O (Figure 6a), only NO_2_ desorption H_2_ is an endothermic process. In the mechanism starting from NO, we see a very small energy difference between the different steps and, after excluding the first most exothermic step, I2, the differences do not exceed 0.30 eV.

This was followed by an analysis of the bond lengths, bond orders, and ionicity of the three key steps in both processes, i.e., 2, 3, and 4. This analysis helps to explain what effect the OH group has on the system and why a Cu-Fe bimetallic catalyst with a hydroxyl group on copper is the most efficient.

**Figure 7:**
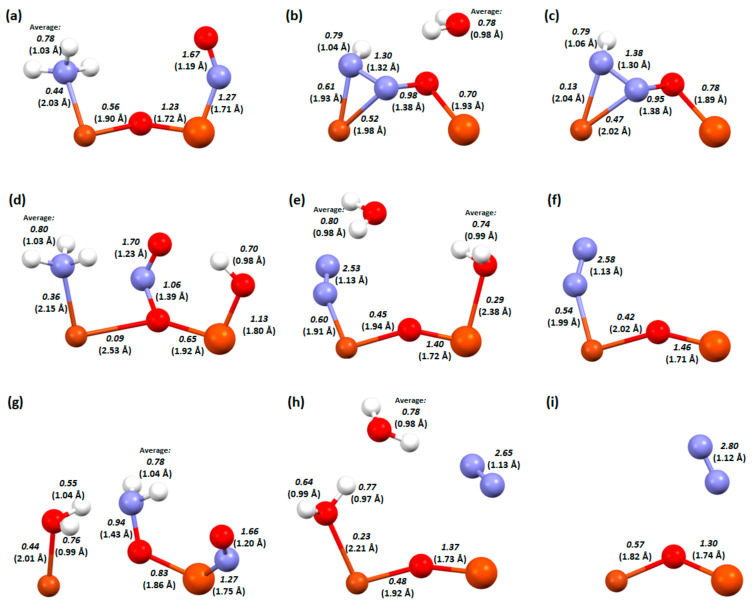
Bond order and length (in bracket) for zeolite FAU structures from the deNOx process: a Cu-O-Fe dimer with an OH group on the bridged oxygen, (**a**) step A3, (**b**) step A4, and (**c**) step A5; a Cu-O-Fe dimer with an OH group on the iron, (**d**) step B2, (**e**) step B3, and (**f**) step B4; and a Cu-O-Fe dimer with an OH group on the copper, (**g**) step C2, (**h**) step C3, and (**i**) step C4.

In Figure 7, we can see the changes that occur between the key steps of the deNOx process on the three versions of the Cu-O-Fe catalyst. In Figure 7a–c, there is a structure where the hydrogen is located on the bridged oxygen.

First, after the conversion and bonding of the two nitrogen atoms, the dimer structure breaks down and the bond between the copper atom and the bridging oxygen breaks down (the transition between these steps is exothermic). Weakening of the bridging oxygen-iron bond (Figure 7a bond order 1.23, Figure 7b bond order 0.70) also occurred. Between the further reaction steps (Figure 7b,c), the bond between the nitrogen atoms and copper weakens (0.61 to 0.13 and 0.52 to 0.47), making the last step, desorption of the nitrogen molecule, exothermic (Figure 1). In the dimer with an OH group on iron, the transformation of the attached adsorbates is followed by an increase in the bond strength in the dimer itself (Cu-O_b_ bond from 0.09 to 0.45, and Fe-O_b_ bond from 0.65 to 1.40). When hydrogen is attached to the OH group, the binding of oxygen to iron is weakened, which significantly simplifies the desorption of the formed water molecule in the next step. Comparing the last key step to the structure where the OH group is attached to the copper dimer, we see that in the first case (Figure 7f), more energy will be required to detach the nitrogen molecule than in the second case, where the nitrogen molecule is already desorbed after the adsorbate transformation step (Figure 7i). This is also confirmed in the description of the mechanisms (Figure 2 and Figure 3). In the last structure, namely the Cu-O-Fe dimer with an OH group on copper (Figure 7g–i) during NH_3_ adsorption into the system, one of the hydrogen atoms is detached to form a water molecule, which is not very strongly bonded to the copper atom (Figure 7g, bond order 0.40). There is also a destabilisation of the dimer system, which may consequently lead to the easier formation of product molecules (the system will move towards reconstitution of the structure and desorption of the adsorbates). In Figure 7h, after the desorption of one of the water and the nitrogen molecules followed by the regeneration of the dimer structure, the oxygen-copper bond order is half weakened (from 0.40 to 0.23).

Figure S2 visualises the charges of the individual atoms.

**Figure 8:**
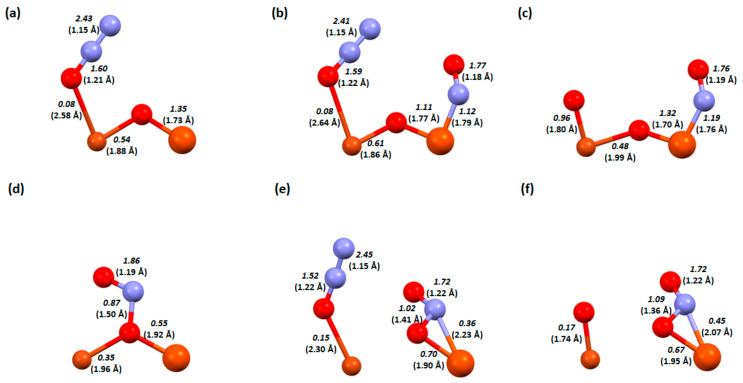
Bond order and length (in bracket) for zeolite FAU structures from the deN_2_O process: a Cu-O-Fe dimer with first desorption of N_2_O. (**a**) Step D2, (**b**) step D3, and (**c**) step D4. Cu-O-Fe dimer with first desorption of NO. (**d**) Step E2, (**e**) step E3, and (**f**) step E4.

In Figure 8, the changes occur in the bond lengths and the orders for the key steps in the deN_2_O process on the Cu-O-Fe dimer.

As previously mentioned, NO adsorption occurred using hydrogen on the bridging oxygen, while N_2_O can desorb itself on the zeolite surface, so an additional hydrogen atom is not needed. Despite the two adsorbates being the same, the order in which they are adsorbed is very important to the arrangement that will form on the catalyst surface. In the adsorption of N_2_O followed by NO, the dimer keeps its structure (Figure 8b), while the adsorption of NO followed by N_2_O disrupts this structure and breaks the copper-oxygen bridge bond (Figure 8e). Although the oxygen–nitrogen bond order is similar in both cases (Figure 8b, 1.59, and Figure 8e, 1.52), the desorption of N_2_ occurs at a lower energy in the second case. This is probably influenced by the fact that the oxygen from N_2_O attaches more strongly to the Cu-O-Fe dimer (bond order 0.15 compared to 0.08 in the first system). In the last step, NO_2_ also desorbs more readily in the second system. Nitrogen is bonded to the iron atom with a bond order of 0.45, while in the first system, it is a bond order of 1.19.

By analysing Figure S3, we can see that all the changes and differences described earlier are also reflected in the ionicity. Of special interest is the difference in the ionicity of the adsorbed NO molecule. In the first case, where the NO molecule attaches to the previously adsorbed N_2_O, the nitrogen atom becomes neutral, while the oxygen atom takes on an ionicity of −0.05 (Figure S3b). In contrast, in the second case, where N_2_O is attached to adsorbed NO (Figure S3e), there is a slight increase in the ionicity of the nitrogen atom (0.16) and a decrease in the ionicity of the oxygen atom (−0.21). This may also be influenced by the migration of NO from the bridging oxygen to the iron atom.

**Figure 9:**
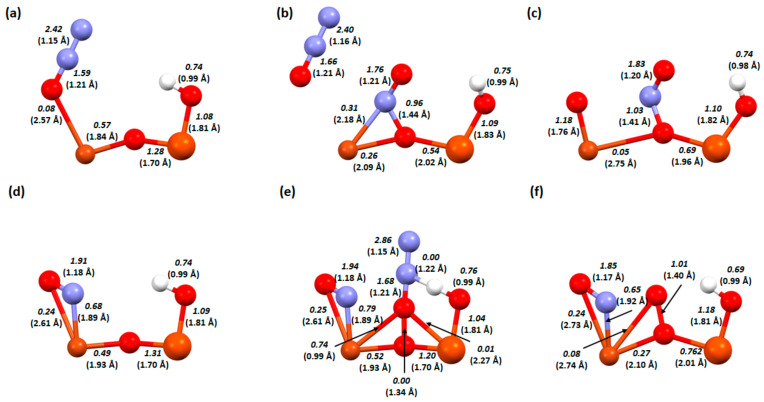
Bond order and length (in bracket) for zeolite FAU structures from the deN_2_O process: a Cu-O-Fe dimer with an OH group on iron with first desorption of N_2_O. (**a**) Step F2, (**b**) step F3, and (**c**) step F4. Dimer Cu-O-Fe with OH group on iron with first desorption of NO. (**d**) Step G2, (**e**) step G3, and (**f**) step G4.

The next system analysed in terms of bond lengths and orders is the one with an OH group on the iron atom (Figure 9). Firstly, when NO attempts to adsorb to the adsorbed N_2_O, it is detached (very weak binding of N_2_O to the dimer—0.08; Figure 8a). In the case of N_2_O adsorption to adsorbed NO, when N_2_O is adsorbed to adsorbed NO, a hydrogen bond is formed with one of the nitrogen atoms in N_2_O and a relatively unusual attachment of N_2_O to the dimer, where the oxygen atom forms bonds with all the atoms in the dimer (Figure 9e). Interestingly, despite these differences in the coadsorption systems, the energy of N_2_ desorption is a little different for the two structures (Figure 5). In contrast, in NO_2_ desorption, more energy is given off to the environment by the first system (Figure 9c), perhaps due to the fact that more bonds need to be broken in the case of the structure in Figure 9f (oxygen is attached to both the copper atom and the bridging oxygen).

In Figure S4, there are changes in ionicity. Interestingly, they are not very noticeable. The only strongly visible change is the large difference in the ionicity of the bridged oxygen after coadsorption (−0.52 Figure S4b, and −0.71 Figure S4e).

**Figure 10:**
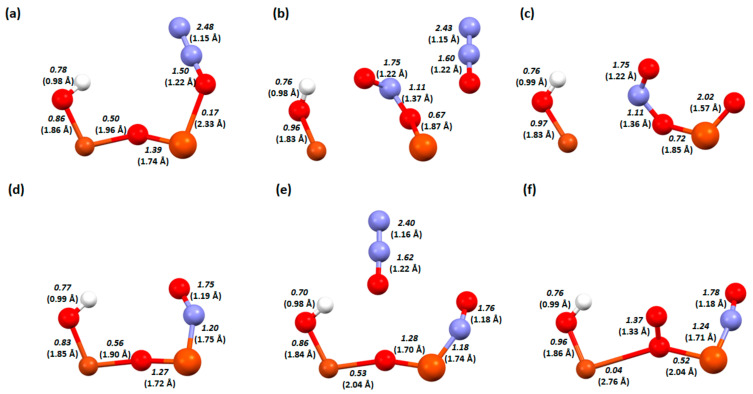
Bond order and length (in bracket) for zeolite FAU structures from the deN_2_O process: a Cu-O-Fe dimer with an OH group on copper with first desorption of N_2_O. (**a**) Step H2, (**b**) step H3, and (**c**) step H4; a Cu-O-Fe dimer with an OH group on the copper with first desorption of NO. (**d**) Step I2, (**e**) step I3, and (**f**) step I4.

The last system for which the bond lengths and bond orders were analysed is the dimer with an OH group on copper (Figure 10). During the coadsorption of NO to N_2_O, similarly to the previous system, the N_2_O molecule is detached (Figure 9b); in addition, the bridge oxygen bond with copper is also broken. In contrast, in a situation where N_2_O attaches to adsorbed NO, no bond is formed with N_2_O (Figure 10e), the oxygen from the nitrogen dioxide molecule only attaches to the bridging oxygen after the desorption of N_2_ (Figure 10f). There is also no destabilisation of the dimer. However, destabilisation of the dimer structure fundamentally affects the energies of the mechanism and makes the coadsorption step in the order of N_2_O then NO strongly exothermic (Figure 6).

In analysing the ionicity of these systems (Figure S5), the bridge oxygen in the dimer shows the most significant changes, particularly after detachment from the copper atom (Figure S5b,c) and after attachment of an oxygen atom from the N_2_O molecule (Figure S5f).

**Figure 11:**
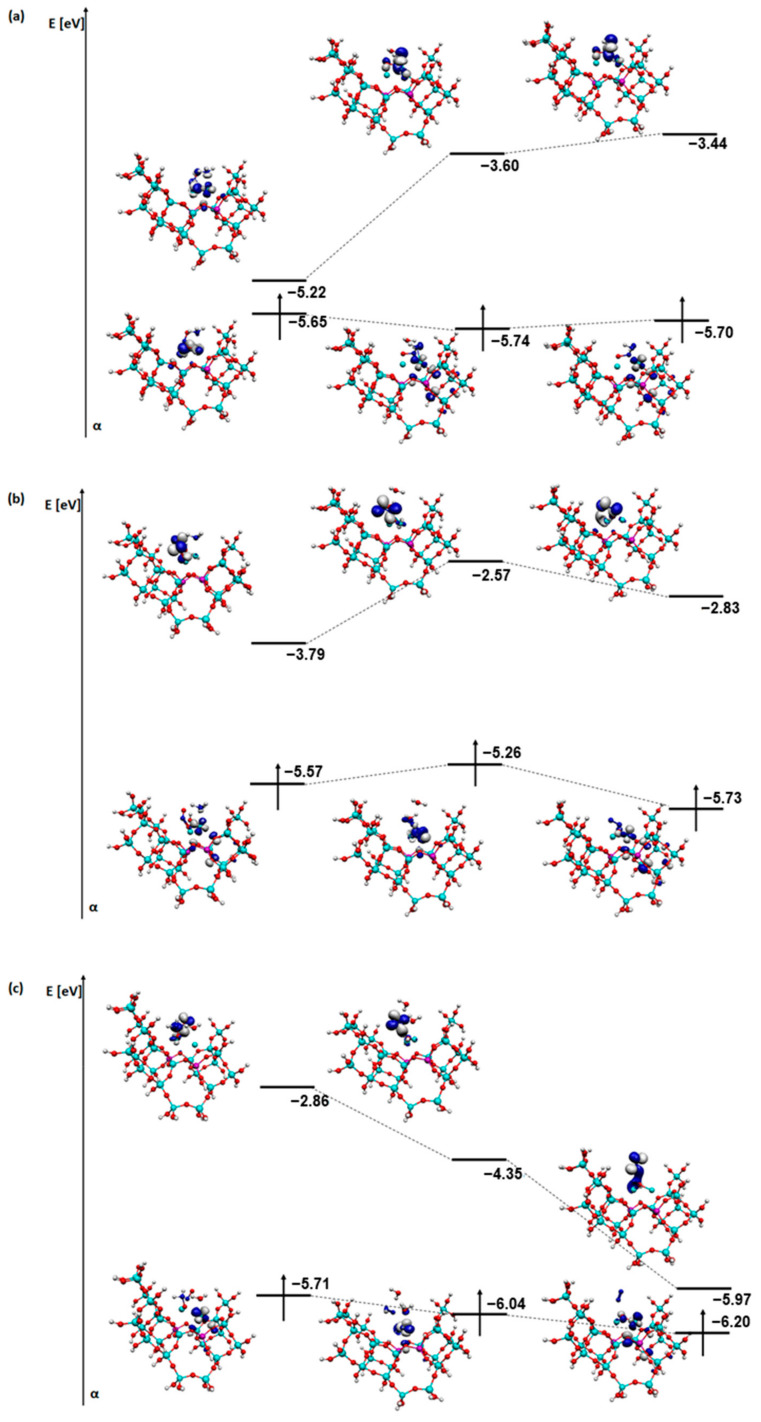
SOMO and LUMO orbitals for the three most important steps in the deNOx mechanism: (**a**) steps A3-A5 for FAU-Cu_OH_Fe, (**b**) steps B3-B5 for FAU-Cu_O_Fe with OH on iron, and (**c**) steps C3-C5 for FAU-Cu_O_Fe with OH on copper.

**Figure 12:**
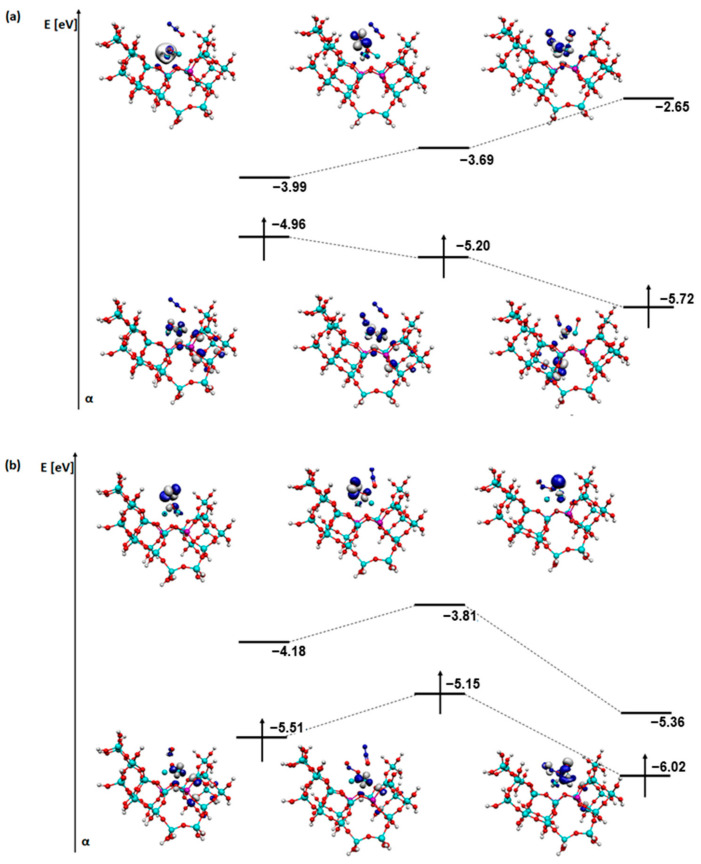
SOMO and LUMO orbitals for the three most important steps in the deN_2_O mechanism: (**a**) steps D2-D4 and (**b**) steps E2-E4 for FAU-Cu_OH_Fe.

**Figure 13:**
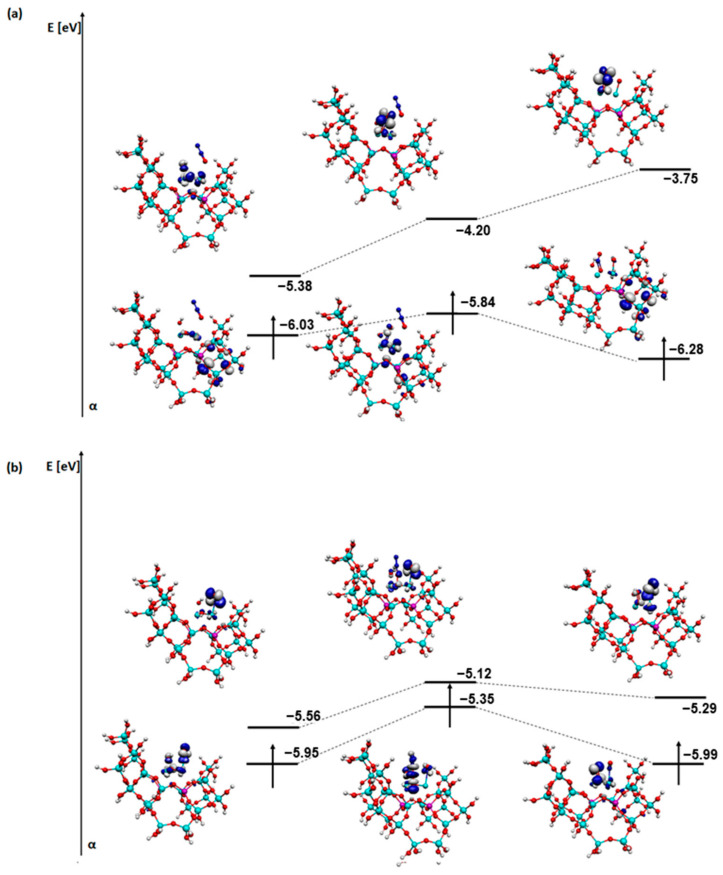
SOMO and LUMO orbitals for the three most important steps in the deN_2_O mechanism: (**a**) steps F2-F4 and (**b**) steps G2-G4 for FAU-Cu_O_Fe with OH on iron.

**Figure 14:**
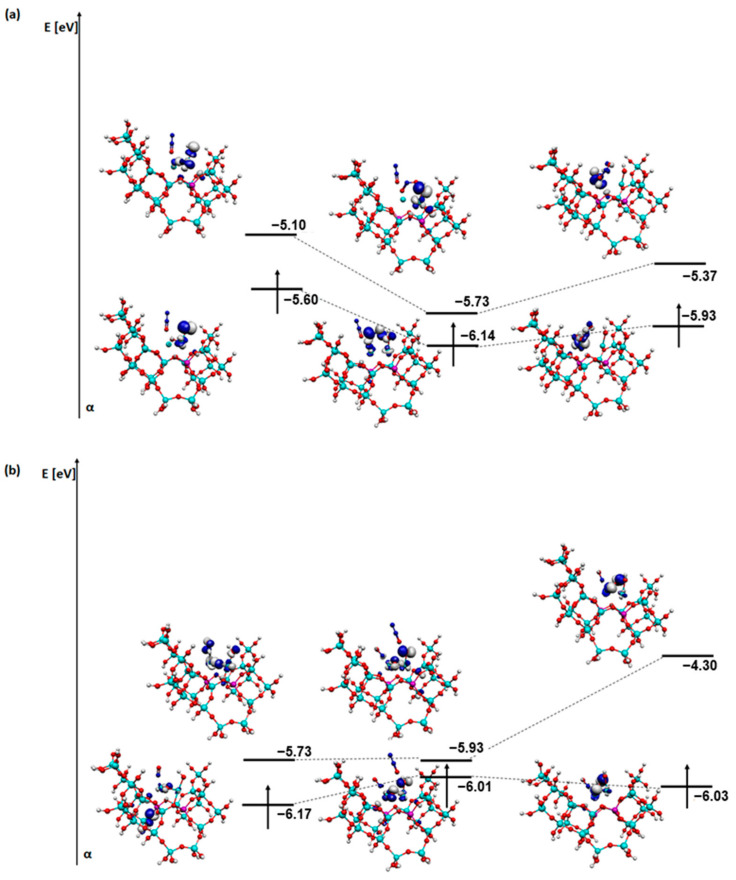
SOMO and LUMO orbitals for the three most important steps in the deN_2_O mechanism: (**a**) steps H2-H4 and (**b**) steps I2-I4 for FAU-Cu_O_Fe with OH on copper.

For a better understanding of the reaction mechanisms, an analysis of the SOMO and LUMO orbitals of the relevant steps of each process was also carried out (Figure 11, Figure 12, Figure 13 and Figure 14). The coadsorption, transformation, and first desorption step (H_2_O or N_2_ depending on the mechanism) were selected.

Several similarities are visible in the visualisation of the orbitals for the deNOx mechanism (Figure 11). Firstly, in all cases, after desorption of a water molecule (or two in the case of structures with an OH group on a metal), the shape of the orbital of both SOMO and LUMO changes slightly. In all stages, the orbitals concentrate particularly near the metal atoms. On the other hand, in the case of the last step and the SOMO orbitals, it can be observed that the orbital also partially covers a fragment of the zeolite structure. Regarding the energy of the orbitals for structures with a bridged OH group, the energy of the LUMO orbital increases, whereas for structures with an OH group on one of the metals, the energy of the LUMO orbitals decreases, especially in the structures with an OH group on copper. The energy of the SOMO orbital stays quite similar in all cases. The mechanism with an OH group on copper is the most energetically favourable in this case, which also coincides with the energy changes in the SOMO and LUMO orbitals.

Several differences appear between the deN_2_O mechanism on a structure with a bridged OH group (Figure 12) and the deNOx mechanism. Firstly, in the SOMO orbitals, the atoms from the zeolite structure had a significant contribution. The shape of the LUMO orbital, which is located on the adsorbates, changes largely. N_2_O does not affect the SOMO or LUMO orbitals at all. The NO molecule is therefore critical, which characterises all systems of the deN_2_O mechanism. Reduction in the energy of the orbitals of both SOMO and LUMO occurred in the deN_2_O mechanism on a structure with a bridged OH group (Figure 12).

The next system analysed is the one with an OH group on an iron atom (Figure 13). In the N_2_O adsorption, the zeolite structure initially takes part in the SOMO orbitals, then disappears and reappears after N_2_ desorption. The shape of the LUMO orbital changes slightly, while its energy increases significantly. In NO adsorption in the first instance, the energy changes between the orbitals are not significant in contrast to the shape. The zeolite does not participate at all in the formation of the orbitals.

The last analysis is of the SOMO and LUMO orbitals for a system with an OH group on copper (Figure 14). As before, N_2_O shows no interference with the orbitals. When the NO adsorption is the first step, there is a reduction in the importance of the SOMO orbital in the zeolite structure (Figure 14b). It is mainly transferred to the adsorbates. The change in the location of the LUMO orbital is particularly noticeable after desorption of the N_2_ molecule in the system as the N_2_O molecule is adsorbed first (Figure 14a). The energy of the SOMO orbitals changes slightly in both cases, while the energy of the LUMO orbital decreases slightly in the first case and increases significantly in the second.

Upon summarising the analysed results, it can be concluded that the most favourable structure for both deNOx and deN_2_O processes is one with an OH group on a copper atom. A system in which N_2_O is adsorbed first is energetically more favourable in the deN_2_O mechanism but this may be difficult to implement due to the higher affinity of NO to the structure. For industrial processes, in order to increase the efficiency of the catalyst, the gas-containing N_2_O molecules would have to be directed to the catalyst first, followed by NO.

## 3. Materials and Methods

### 3.1. Computational Details

The ab initio density functional theory (DFT) method was used to calculate the electron structure of the presented clusters using the StoBe program [40]. The non-local generalised gradient corrected functionals according to Perdew, Burke, and Ernzerhof (RPBE) [41,42] were used to take account of the electron exchange and correlation. Kohn–Sham orbitals were represented by linear combinations of atomic orbitals (LCAOs) using contracted Gaussian basis sets for atoms [43]. Mulliken populations [44] and Mayer bond order factors [45,46] were used to precisely analyse the electron structure of the clusters.

Double valence zeta polarisation (DZVP) functional bases were used for Si and Al (6321/521/1), Cu and Fe (63321/531/311), O and N (621/41/1), and H (41) orbital basis sets [47]. Auxiliary functional bases were also used to adjust the electron density and the exchange potential of the correlation of individual atoms: Si and Al (5,4;5,4), Cu and Fe (5,5;5,5), O and N (4,3;4,3), and H (4,0;4,0).

The calculations took into account the structures with the lowest energy (for each structure, all probable multiplicities were calculated—Tables S1 and S2).

The energy difference between the different stages of the mechanism was calculated as follows:

$$E_{diff}=E_{next\_step}-E_{previous\_step}\pm E_{substrates/products}\;\left[\mathrm{eV}\right]$$

Full descriptions of the formulae are included in the Supplementary Materials.

### 3.2. Geometrical Models

The structure of the FAU zeolite was taken from the Database of Zeolite Structure [48]. A single crystal unit cell contains 706 atoms, the cubic phase of the FAU zeolite framework type is characterised by the space group Fd-3m (# 227) with lattice constants a = b = c = 24.3450 Å.

To create the cluster required for the calculations, a fragment of crystal containing 24T positions with an Al_2_Si_22_O_66_H_36_ structure was cut out. A series of zeolites with Si/Al ratios in the range of 2.6–30 are commercially available [49]. In our structure, the active center was formed by exchanging two silicon atoms for two aluminum atoms. The Si/Al ratio in the cluster structure is 11 (22 silicon atoms and 2 aluminum atoms). The broken bonds were saturated with a point charge, which was represented by a hydrogen atom. The hydrogen was placed at a distance of 0.97 Å in a direction consistent with that presented by the broken Si-O bond. The fragment contained important active sites for the catalyst including two aluminum atoms. This structure was successfully used in earlier studies [33].

**Figure 15:**
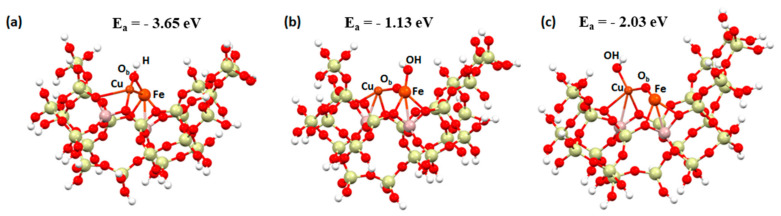
Zeolite FAU structure used to calculate a (**a**) Cu-O_b_-Fe dimer and an OH group on the oxygen bridge, (**b**) Cu-O_b_-Fe dimer with an OH group on the Cu, and (**c**) Cu-O_b_-Fe dimer with an OH group on the Fe. Adsorption energy of the OH group in the above structure.

Figure 15 shows the structures that were used to perform the selective catalytic reduction in both the deNOx and deN_2_O processes. The structures were formed by adsorption of dimers near the active site, which is represented by two aluminum atoms.

To simulate the mechanism, three structures were designed: an FAU zeolite cluster with a Cu-Fe bimetallic dimer and a hydroxyl group on bridged oxygen (Figure 15a), an FAU zeolite cluster with a Cu-Fe bimetallic dimer and a hydroxyl group on iron (Figure 15b), and an FAU zeolite cluster with a Cu-Fe bimetallic dimer and a hydroxyl group on copper (Figure 15c).

The distribution of ionicity and bond lengths are shown in Figure S1 in Supplementary Materials.

## 4. Conclusions

To summarise the presented analyses, some essential findings can be confirmed. First, the presence of an OH group on one of the metals in the dimer improves the energetics of the reaction mechanism and the way NO and NH_3_ molecules react with each other in the deNOx process. We can find confirmation of this in the analysis of the bond orders and lengths, ionicity, and orbitals. The order in which NO and N_2_O molecules are adsorbed has a significant influence on the deN_2_O process. For industrial processes, it is suggested to introduce N_2_O first and then NO into the catalytic system. For both the deNOx and deN_2_O mechanisms, the FAU-Cu-O-Fe structure with an OH group on copper shows the highest efficiency. The analyses presented here show that molecular modelling makes it possible to verify the different behaviours of adsorbates during industrial processes, which enables an in-depth understanding of the reactions that occur and also leads to the possibility of proposing changes in the catalytic system.

## Data Availability

Data are contained within the article or Supplementary Materials.

## Supplementary Materials

The following are available online at https://www.mdpi.com/article/10.3390/molecules29102329/s1, Figure S1. Bond order and length (in brackets) for zeolite FAU structures. (a) Cu-O-Fe dimer with an OH group on the bridged oxygen, (b) Cu-O-Fe dimer with an OH group on the iron, (c) Cu-O-Fe dimer with an OH group on the copper and ionicity for zeolite FAU structures: (d) Cu-O-Fe dimer with an OH group on the bridged oxygen, (e) Cu-O-Fe dimer with an OH group on the iron, and (f) Cu-O-Fe dimer with an OH group on the copper. Figure S2. Ionicity for zeolite FAU structures from the deNOx process: Cu-O-Fe dimer with an OH group on the bridged oxygen. (a) Step A3, (b) step A4, and (c) step A5; Cu-O-Fe dimer with an OH group on iron. (d) Step B2, (e) step B3, and (f) step B4; Cu-O-Fe dimer with an OH group on copper—Step C2, (g) step C3, and (h) step C4. Figure S3. Ionicity for zeolite FAU structures from thedeN_2_O process: dimer Cu-O-Fe with first desorption of N_2_O. (a) Step D2, (b) step D3, and (c) step D4; Cu-O-Fe dimer with first desorption of NO. (d) Step E2, (e) step E3, and (f) step E4. Figure S4. Ionicity for zeolite FAU structures from the deN_2_O process: Cu-O-Fe dimer with an OH group on iron with first desorption of N_2_O. (a) Step F2, (b) step F3, and (c) step F4; Cu-O-Fe dimer with an OH group on iron with first desorption of NO. (d) Step G2, (e) step G3, and (f) step G4. Figure S5. Ionicity for zeolite FAU structures from the deN_2_O process: Cu-O-Fe dimer with an OH group on copper with first desorption of N_2_O. (a) Step H2, (b) step H3, and (c) step H4; Cu-O-Fe dimer with an OH group on the copper with first desorption of NO. (d) Step I2, (e) step I3, and (f) step I4. Table S1. Energies for different structures and considered multiplicities for the deNOx process. Formulas for energy difference between stages in mechanism with bridged OH group. Table S2. Energies for different structure and considered multiplicities for deN2O process.
