# Supplementary material for: Mechanisms for deNOx and deN2O Processes on FAU Zeolite with a Bimetallic Cu-Fe Dimer in the Presence of a Hydroxyl Group—DFT Theoretical Calculations
Source: Molecules. 2024 May 15;29(10):2329. doi: 10.3390/molecules29102329 (PMC11123728; doi:10.3390/molecules29102329)
Supplement: Supplementary file 1 [file molecules-29-02329-s001.zip › molecules-2995791-supplementary.pdf]

molecules

MDPI

Supplementary Informations of Article

Mechanisms for deNOx and deN2O Processes on FAU Zeolite with a Bimetallic Cu-Fe Dimer in the Presence of a Hydroxyl Group—DFT Theoretical Calculations

Izabela Kurzydym 1,2 and Izabela Czekaj 3,*

1 Faculty of Chemistry, University of Warsaw, ul. Pasteura 1, 02-093 Warsaw, Poland; chemia@chem.uw.edu.pl

2 Biological and Chemical Research Center, University of Warsaw, ul. Żwirki i Wigury 101, 01-224 Warsaw, Poland

3 Faculty of Chemical Engineering and Technology, Cracow University of Technology, Warszawska 24, 31-155 Kraków, Poland

* Correspondence: izabela.czekaj@pk.edu.pl

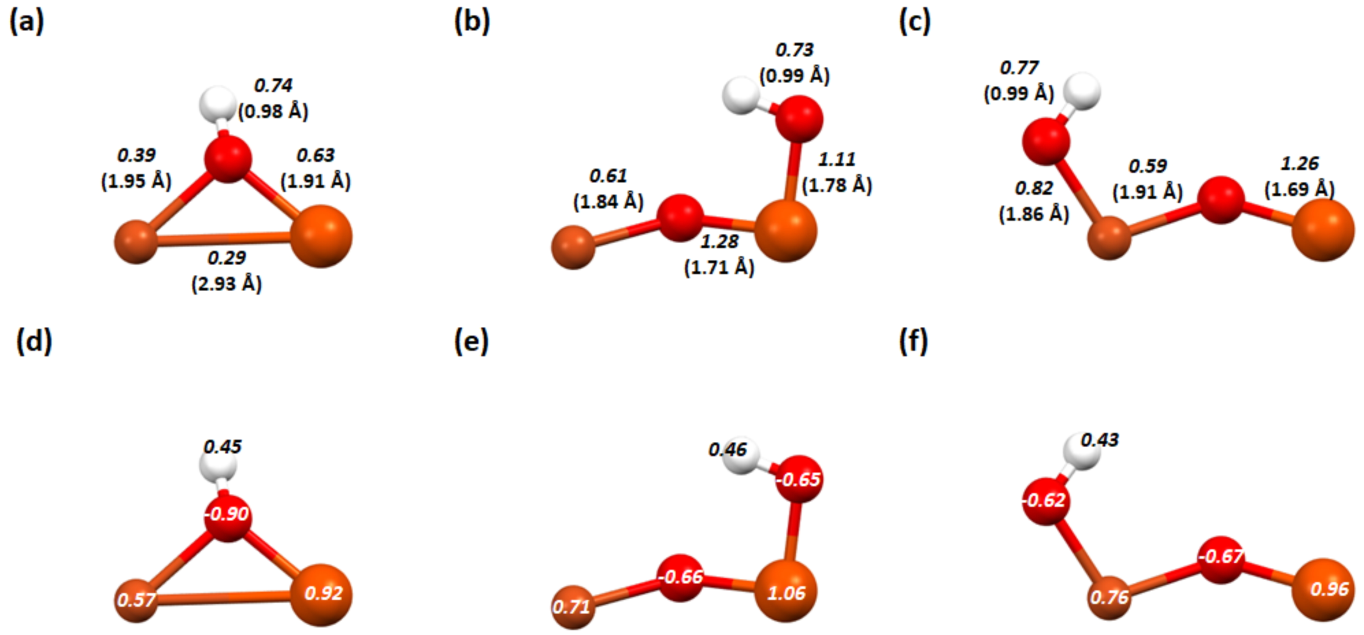

Figure S1. Bond order and lenght (in brackets) for zeolite FAU structures a) Cu-O-Fe dimer with OH group on bridged oxygen, b) Cu-O-Fe dimer with OH group on iron, c) Cu-O-Fe dimer with OH group on copper and ionicity for zeolite FAU structures: d) Cu-O-Fe dimer with OH group on bridged oxygen, e) Cu-O-Fe dimer with OH group on iron, f) Cu-O-Fe dimer with OH group on copper.

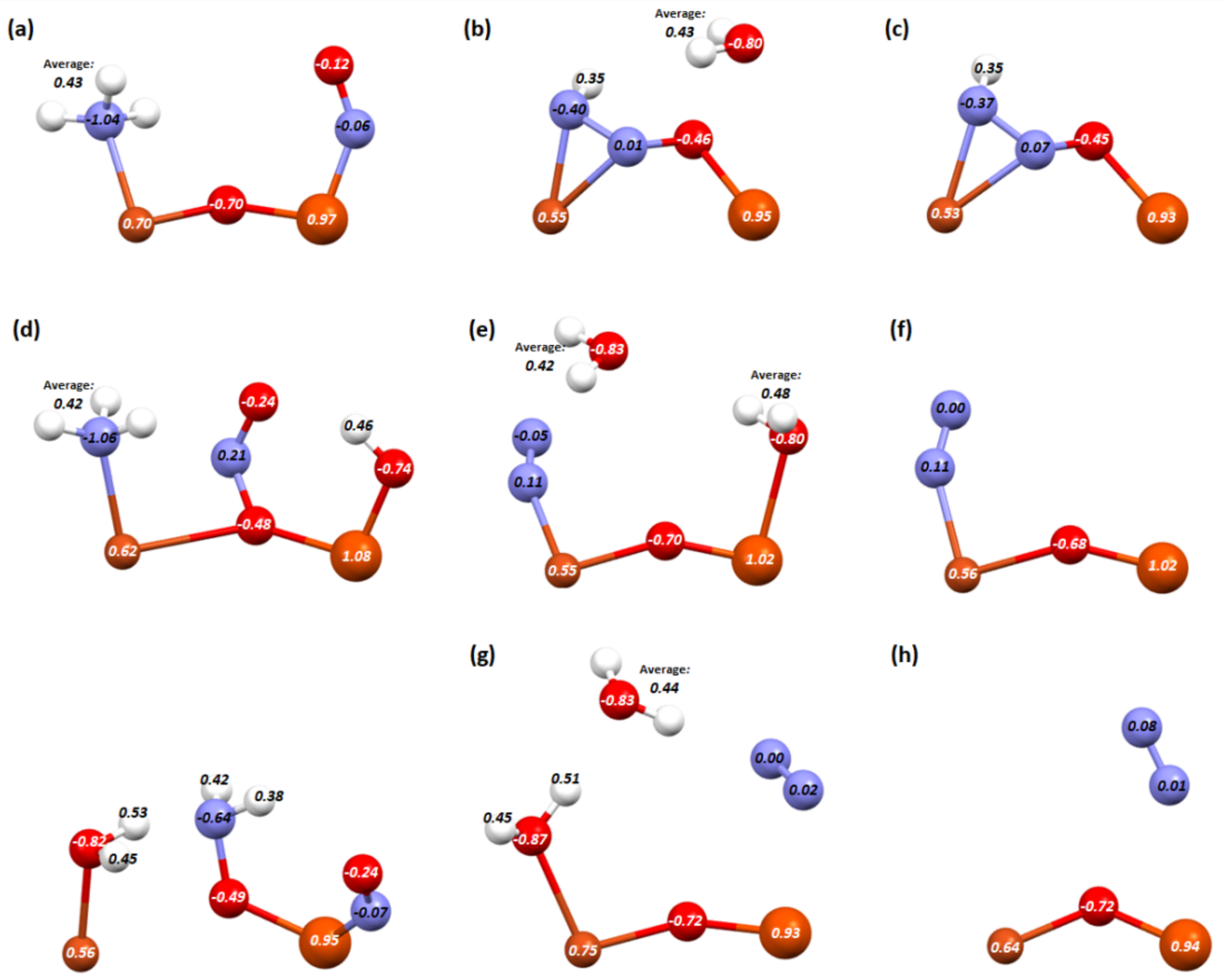

Figure S2. Ionicity for zeolite FAU structures from deNOx process: dimer Cu-O-Fe with OH group on bridged oxygen – a) step A3, b) step A4, c) step A5; dimer Cu-O-Fe with OH group on iron – d) step B2, e) step B3, f) step B4; dimer Cu-O-Fe with OH group on copper – step C2, g) step C3, h) step C4.

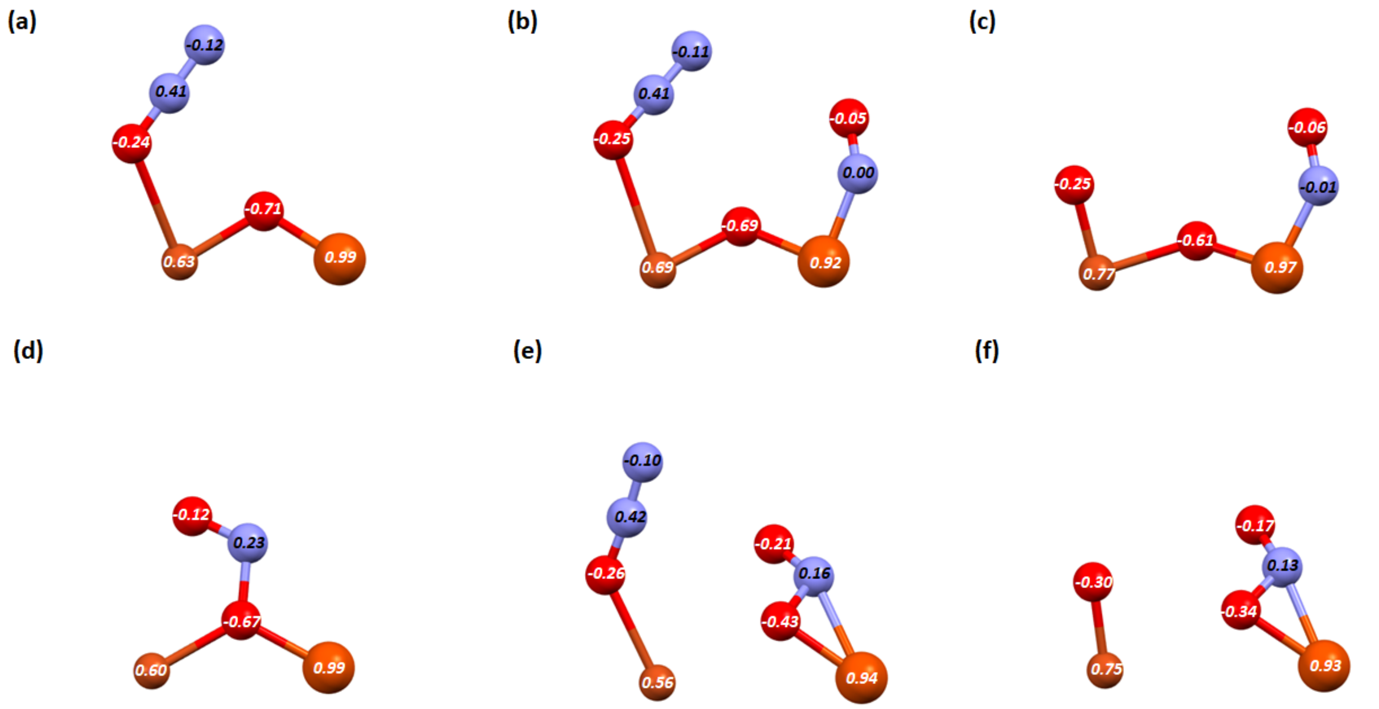

Figure S3. Ionicity for zeolite FAU structures from deN2O process: dimer Cu-O-Fe with first desorption of N2O – a) step D2, b) step D3, c) step D4; dimer Cu-O-Fe with first desorption of NO – d) step E2, e) step E3, f) step E4.

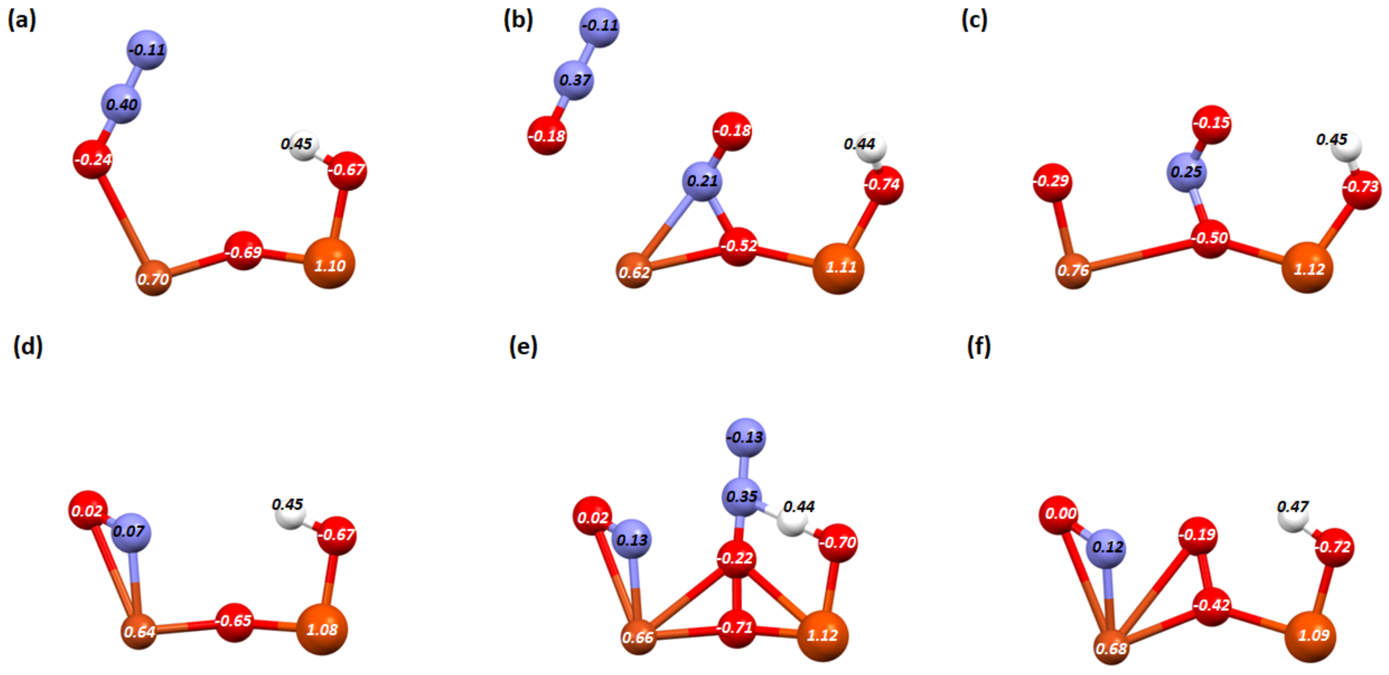

Figure S4. Ionicity for zeolite FAU structures from deN2O process: dimer Cu-O-Fe with OH group on iron with first desorption of N2O – a) step F2, b) step F3, c) step F4; dimer Cu-O-Fe with OH group on iron with first desorption of NO – d) step G2, e) step G3, f) step G4.

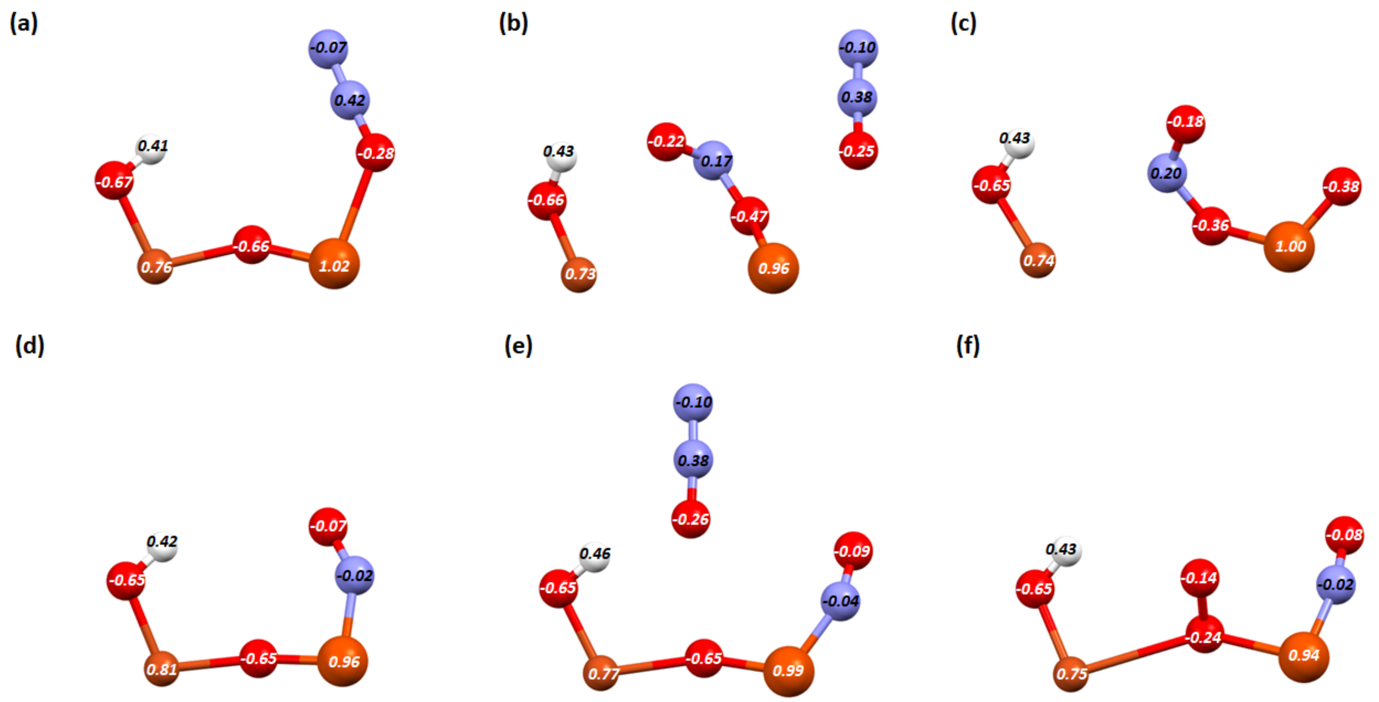

Figure S5. Ionicity for zeolite FAU structures from deN2O process: dimer Cu-O-Fe with OH group on copper with first desorption of N2O – a) step H2, b) step H3, c) step H4; dimer Cu-O-Fe with OH group on copper with first desorption of NO – d) step I2, e) step I3, f) step I4.

Table S1. Energies for different structure and considered multiplicities for deNOx process.

Structure	Multiplicity	Energy [H]
A1	1	-14593.383
	3	-14593.382
	5	-14593.417
	7	-14593.324
	9	-14593.195
A2	1	-14722.707
	3	-14722.715
	5	-14722.739
	7	-14722.708
	9	-14722.609
A3	1	-14779.355
	3	-14779.376
	5	-14779.364
	7	-14779.323
A4	1	-14779.291
	3	-14779.279
	5	-14779.310
	7	-14779.296

	9	-14779.171			
A5	1	-14702.853			
	3	-14702.791			
	5	-14702.875			
	7	not converged			
	9	-14702.710			
Structure	Multiplicity	Energy [H]	Structure	Multiplicity	Energy [H]
B1	2	-14592.774	C1	2	-14592.774
	4	-14592.780		4	-14592.780
	6	-14592.780		6	-14592.780
	8	-14592.675		8	-14592.675
B2	1	-14668.610	C2	1	-14668.543
	3	-14668.610		3	-14668.591
	5	-14668.624		5	-14668.587
	7	-14668.602		7	-14668.586
	9	-14668.462		9	-14668.473
B3	2	-14855.155	C3	2	-14855.140
	4	-14855.158		4	-14855.157
	6	-14855.169		6	-14855.109
	8	-14855.151		8	-14855.066
	10	-14854.967		10	-14855.030
B4	2	not converged	C4	2	-14855.119
	4	-14855.223		4	-14855.068
	6	-14855.226		6	-14855.216
	8	-14855.075		8	-14854.995
	10	-14854.977		10	-14854.970
B5	2	-14702.311	C5	2	-14702.315
	4	-14702.334		4	-14702.325
	6	-14702.335		6	-14702.333
	8	-14702.239		8	-14702.136
	10	-14702.084		10	-14702.023

Table S2. Energies for different structure and considered multiplicities for deN2O process.

Structure	Multiplicity	Energy [H]	Structure	Multiplicity	Energy [H]
D1	2	-14592.774	E1	1	-14593.383
	4	-14592.780		3	-14593.382
	6	-14592.780		5	-14593.417
	8	-14592.675		7	-14593.324
D2	2	-14777.477		9	-14593.195
	4	-14777.491	E2	1	-14722.707

	6	-14777.503		3	-14722.715
	8	-14777.401		5	-14722.739
	10	-14777.351		7	-14722.708
D3	1	-14907.470		9	-14722.609
	3	-14907.478	E3	1	not converged
	5	-14907.478		3	not converged
	7	-14907.431		5	-14907.434
	9	-14907.365		7	not converged
D4	1	not converged		9	not converged
	3	-14797.885	E4	1	-14797.828
	5	-14797.892		3	-14797.882
	7	-14797.876		5	-14797.864
	9	-14797.852		7	-14797.877
				9	-14797.831

Structure	Multiplicity	Energy [H]	Structure	Multiplicity	Energy [H]
F1	1	-14668.610	G1	1	-14668.610
	3	-14668.610		3	-14668.610
	5	-14668.624		5	-14668.624
	7	-14668.602		7	-14668.602
	9	-14668.462		9	-14668.462
F2	1	-14853.318	G2	2	-14798.560
	3	-14853.332		4	-14798.571
	5	-14853.353		6	-14798.575
	7	-14853.332		8	-14798.544
	9	-14853.288		10	-14798.448
F3	2	-14983.280	G3	2	-14983.269
	4	-14983.283		4	-14983.287
	6	-14983.287		6	-14983.298
	8	not converged		8	-14983.260
	10	-14983.208		10	-14983.164
F4	2	-14873.691	G4	2	-14873.712
	4	not converged		4	-14873.727
	6	-14873.709		6	-14873.733
	8	-14873.719		8	-14873.724
	10	-14873.635		10	-14873.649
	12	-14873.477			

Structure	Multiplicity	Energy [H]	Structure	Multiplicity	Energy [H]

H1	1	-14668.543	I1	1	-14668.543
	3	-14668.591		3	-14668.591
	5	-14668.587		5	-14668.587
	7	-14668.586		7	-14668.586
	9	-14668.473		9	-14668.473
H2	1	-14853.289	I2	2	-14798.554
	3	-14853.304		4	-14798.558
	5	-14853.308		6	-14798.554
	7	-14853.305		8	-14798.514
	9	-14853.277		10	-14798.434
H3	2	-14983.213	I3	2	-14983.267
	4	-14983.259		4	not converged
	6	-14983.252		6	not converged
	8	-14983.214		8	-14983.182
	10	-14983.173		10	-14983.162
H4	2	-14873.750	I4	2	not converged
	4	-14873.748		4	-14873.730
	6	-14873.742		6	-14873.732
	8	-14873.718		8	-14873.631
	10	-14873.621		10	-14873.593

Energy difference between stages in mechanism with bridged OH group were calculated as follows:

A. Energy for deNOx process on Cu-O-Fe dimer with OH group on bridged oxygen:

1. Energy difference between stage A2 and A1:
$$E_{diff} = E_{A2} – E_{A1} – 2E_{NO2} + E_{HNO3} \text{ [eV]}$$
2. Energy difference between stage A3 and A2:
$$E_{diff} = E_{A3} – E_{A2} – E_{NH3} \text{ [eV]}$$
3. Energy difference between stage A4 and A3:
$$E_{diff} = E_{A4} – E_{A3} \text{ [eV]}$$
4. Energy difference between stage A5 and A4:
$$E_{diff} = E_{A5} – E_{A4} + E_{H2O} \text{ [eV]}$$
5. Energy difference between stage A1 and A5:
$$E_{diff} = E_{A1} – E_{A5} + E_{N2} \text{ [eV]}$$

B. Energy for deNOx process on Cu-O-Fe dimer with OH group on one of the metal atom in dimer:

1. Energy difference between stage B2 and B1 (same for C diagram):
$$E_{diff} = E_{B2} – E_{B1} – E_{NO} – E_{NH3} \text{ [eV]}$$
2. Energy difference between stage B3 and B2 (same for C diagram):
$$E_{diff} = E_{B3} – E_{B2} \text{ [eV]}$$
3. Energy difference between stage B4 and B3 (same for C diagram):
$$E_{diff} = E_{B4} – E_{B3} + 2E_{H2O} \text{ [eV]}$$
4. Energy difference between stage B5 and B4 (same for C diagram):
$$E_{diff} = E_{B5} – E_{B4} + E_{N2} \text{ [eV]}$$

C. Energy for deN$_2$O process on Cu-O-Fe dimer with OH group on bridged oxygen (start with N$_2$O):

1. Energy difference between stage D2 and D1 (same for F and H diagrams):
$$E_{diff} = E_{D2} - E_{D1} - E_{N2O} \text{ [eV]}$$
2. Energy difference between stage D3 and D2 (same for F and H diagrams):
$$E_{diff} = E_{D3} - E_{D2} - E_{NO} \text{ [eV]}$$
3. Energy difference between stage D4 and D3 (same for F and H diagrams):
$$E_{diff} = E_{D4} - E_{D3} + E_{N2O} \text{ [eV]}$$
4. Energy difference between stage D1 and D4 (same for F and H diagrams):
$$E_{diff} = E_{D1} - E_{D4} + E_{NO2} \text{ [eV]}$$

D. Energy for deN2O process on Cu-O-Fe dimer with OH group on bridged oxygen (start with NO):

1. Energy difference between stage E2 and E1 (same for G and I diagrams):
$$E_{diff} = E_{E2} - E_{E1} - E_{NO} - E_{NH3} \text{ [eV]}$$
2. Energy difference between stage E3 and E2 (same for G and I diagrams):
$$E_{diff} = E_{E3} - E_{E2} - E_{N2O} \text{ [eV]}$$
3. Energy difference between stage E4 and E3 (same for G and I diagrams):
$$E_{diff} = E_{E4} - E_{E3} + E_{N2} \text{ [eV]}$$
4. Energy difference between stage E5 and E4 (same for G and I diagrams):
$$E_{diff} = E_{E5} - E_{E4} + E_{NO2} \text{ [eV]}$$
